# Effects of age and axial length on choroidal stratified structure in normal eyes

**DOI:** 10.1038/s41598-024-52627-x

**Published:** 2024-01-30

**Authors:** Yuki Ito, Hiroaki Endo, Satoru Kase, Mitsuo Takahashi, Shozo Sonoda, Tomonori Sakoguchi, Taiji Sakamoto, Susumu Ishida, Manabu Kase

**Affiliations:** 1 Department of Ophthalmology, Teine Keijinkai Hospital, 1-12 Maeda, Teine-Ku, Sapporo, 006-8555 Japan; 2 Department of Ophthalmology, Faculty of Medicine and Graduate School of Medicine, Hokkaido University, Sapporo, Japan; 3 Department of Ophthalmology, Kagoshima University Graduate School of Medical and Dental Sciences, Kagoshima, Japan

**Keywords:** Biomarkers, Medical research

## Abstract

To quantify the choroidal structures of normal eyes by optical coherence tomography (OCT)-based binarization and evaluate the relationships among age, refractive power, and ocular axial length. This was a retrospective observational study. One hundred and eighty nine eyes of 189 subjects without ocular diseases were examined by enhanced depth imaging (EDI)-OCT. A choroidal OCT horizontal image with a width of 1500 μm centered on the fovea was binarized. The lumen, stroma, and total choroidal area in the choriocapillaris (CC), Sattler’s layer (SL), and Haller's layer (HL) were measured, and the ratio of the luminal area to total choroidal area (L/C ratio) was calculated. Multiple regression analysis was performed for choroidal parameters in each choroidal layer and for age, refractive power, and ocular axial length. Multiple regression analysis showed that an older age was significantly correlated with a lower choroidal area and the L/C ratio in all choroidal layers (each *P* < 0.05). A Long axial length was significantly associated with lower SL and HL (*P* < 0.05), but not with refractive power. In the choroid of normal eyes, age-related decreases in the choroidal area and L/C ratio were associated with all choroidal layers, and elongation of the axial length was associated with thinning of SL and HL.

## Introduction

The choroid is histologically classified by vascular structures into three layers: Choriocapillaris (CC), Sattler's layer (SL), and Haller's layer (HL), which are adjacent to the retinal pigment epithelium on the retinal side^1^.

Until now, choroidal vasculatures have been evaluated by clinical and histological methods. Indocyanine green angiography has been a conventional method for observing the choroidal vasculature in human eyes, but it is invasive due to the use of contrast media and cannot be frequently performed. Recent advances in optical coherence tomography (OCT) have made it possible to noninvasively and quantitatively evaluate the choroidal thickness. With the OCT B-scan, CC and SL can be evaluated as the inner choroidal layer and HL as the outer choroidal layer^2,3^. However, it is difficult to detect the boundary between the three layers on OCT images, and manual detection is not highly reproducible due to significant inter-examiner bias. On the other hand, although it is possible to identify organic changes such as neovascularization and inflammation in the choroidal structure of enucleated or autopsied eyes^4^, it is difficult to accurately assess the thickness and structure of the three layers due to the collapse of the eyeball caused by ocular artery transection^5^. In addition, since eyeball enucleation is performed only in limited cases, structural analysis of the normal choroid by enrolling many cases is impossible.

For this reason, it has been challenging to evaluate the choroidal vasculature in detail and with high reproducibility. Recently, Sonoda et al. developed a new software, named “Kago-Eye2”, which can automatically calculate the choroidal vascular structure using the OCT B-scan, and facilitate quantitative analyses^6^. In fact, with this automatic analysis software, it is possible to convert any range of the choroid into binarization, and immediately analyze the thickness, vascular lumen, and extravascular areas. Kago-Eye2 showed favorable inter-observer reproducibility in choroidal structure analysis^6^. However, little is known about the details of the highly reliable choroidal layer structures in normal eyes.

It has been reported that the choroid thins with aging, myopia, or axial elongation^7^, but it is not clear how each stratification is involved. In this study, we quantified the choroidal structure of normal eyes by application of the new binarization method “Kago-Eye2”, and evaluated the relationship with age, refractive power, and axial length.

## Results

As shown in Table 1, the inter-rater reliability values for both the L/C ratio and LA in CC, SL, and HL ranged from ICC 0.8 to 0.9 for Spearman's rank correlation coefficient. In addition, neither a fixed nor systematic bias significantly affected the results in this study, confirming high reliability and reproducibility (Table 1). The results of the choroidal stratification are shown in Table 2. CCT was 259.4 ± 86.9, 16.2 ± 2.6, 72.7 ± 19.4, and 170.4 ± 72.0 μm for the entire choroid, CC, SL, and HL, respectively. The areas of the entire choroid, CC, SL, and HL were 0.39 ± 0.13, 0.04 ± 0.01, 0.09 ± 0.03, and 0.26 ± 0.11 mm^2^, respectively. The L/C ratios (%) were 66.5 ± 3.4, 79.0 ± 6.3, 67. 6± 7.3, and 63.3 ± 6.6 for the whole choroid, CC, SL, and HL, respectively. The L/C ratio was highest in CC and decreased in order of SL and HL (*P* < 0.01).

**Table 1:** Inter-examiner reliability of choroidal segmentation for study subjects.

Variables						Bland–Altman analysis			
		Relative reliability				Fixed bias		Proportional bias	
		ICC (single)	*P* value	ICC (mean)	*P* value	CI 95%	*P* value	*R*	*P* value
Choriocapillaris (CC)	CC area	0.942	< 0.01	0.970	< 0.01	− 254 ~ 516	0.50	− 0.01	0.90
	L/C ratio	0.991	< 0.01	0.994	< 0.01	− 0.12 ~ 0.12	0.99	− 0.06	0.45
Sattler’s layer (SL)	SL area	0.887	< 0.01	0.913	< 0.01	− 2071 ~ 2074	0.99	0.03	0.73
	L/C ratio	0.960	< 0.01	0.980	< 0.01	− 0.26 ~ 0.34	0.78	− 0.06	0.42
Haller’s layer (HL)	HL area	0.990	< 0.01	0.995	< 0.01	− 1433 ~ 2993	0.49	− 0.00	0.99
	L/C ratio	0.991	< 0.01	0.995	< 0.01	− 0.12 ~ 0.14	0.90	0.08	0.29

*ICC* intra-class coefficient, *CI 95%* 95% confidence interval, *L/C ratio* luminal/choroidal ratio.

**Table 2:** Choroidal layers and L/C ratio in the analysis range of the study subjects.

Variables	Analysis range 1500 μm		CCT, μm
	Area, mm^2^	L/C ratio, %	
Total choroid	0.39 ± 0.13	66.5 ± 3.4	259.4 ± 86.9
Choriocapillaris	0.04 ± 0.01	79.0 ± 6.3	16.2 ± 2.6
Sattler’s layer	0.09 ± 0.03	67.6 ± 7.3	72.7 ± 19.4
Haller’s layer	0.26 ± 0.11	63.3 ± 6.6	170.4 ± 72.0

*L/C ratio* luminal/choroidal ratio, *CCT* central choroidal thickness.

Next, Table 3 shows the results of multiple regression analysis looking at each stratified parameter with age, refractive power, and ocular axial length. Older age was associated with thinning of all choroidal sublayers. Refractive power did not show consistent associations across all sublayers. Longer axial length was associated with decreased choroidal parameters in SL and HL. However, no relationship between axial length and CC was observed.

**Table 3:** Multiple regression analysis of age and axial length for choroidal parameters of normal eyes.

Variables	Analysis range 1500 μm					
	Age		SE		AL	
	*β*	*P* value	*β*	*P* value	*β*	*P* value
Total choroid						
Choroidal area	** − 0.48**	** < 0.01**	0.09	0.38	** − 0.57**	** < 0.01**
Luminal area	** − 0.47**	** < 0.01**	0.11	0.32	** − 0.56**	** < 0.01**
Stromal area	** − 0.46**	** < 0.01**	0.06	0.59	** − 0.58**	** < 0.01**
L/C ratio	** − 0.25**	** < 0.01**	0.13	0.32	− 0.22	0.10
Choriocapillaris						
Choroidal area	** − 0.30**	** < 0.01**	0.15	0.23	− 0.23	0.09
Luminal area	** − 0.44**	** < 0.01**	**0.24**	**0.052**	− 0.18	0.14
Stromal area	**0.29**	** < 0.01**	− 0.17	0.20	− 0.16	0.23
L/C ratio	** − 0.50**	** < 0.01**	**0.25**	**<0.05**	− 0.02	0.87
Sattler’s layer						
Choroidal area	** − 0.45**	** < 0.01**	0.13	0.28	** − 0.38**	** < 0.01**
Luminal area	** − 0.49**	** < 0.01**	0.08	0.51	** − 0.39**	** < 0.01**
Stromal area	** − 0.27**	** < 0.01**	0.20	0.12	**− 0.27**	**<0.05**
L/C ratio	** − 0.20**	** < 0.05**	− 0.09	0.49	− 0.05	0.69
Haller’s layer						
Choroidal area	** − 0.43**	** < 0.01**	0.07	0.60	** − 0.57**	** < 0.01**
Luminal area	** − 0.40**	** < 0.01**	0.09	0.45	** − 0.56**	** < 0.01**
Stromal area	** − 0.56**	** < 0.01**	0.02	0.86	** − 0.56**	** < 0.01**
L/C ratio	** − 0.20**	** < 0.01**	0.06	0.63	** − 0.42**	** < 0.01**

Significant values are in bold.

*L/C ratio* luminal/choroidal ratio, *SE* spherical equivalent, *AL* axial length.

## Discussion

This study demonstrated that choroidal areas were the greatest in HL followed by SL and CC, and the L/C ratio was conversely the highest in CC followed by SL and HL. Furthermore, decreased choroidal parameters were associated with older age and a longer axial length.

Histologically, the choroid is composed of vascular and stromal tissues. The vessels are made up of CC and parenchyma (SL and HL), and the stroma contains melanocytes, smooth muscles, neurons, and connective tissues^8^. Previously, Sonoda et al. examined 180 eyes that were binarized using EDI-OCT with the 7500-μm range, and multivariate analysis showed that both age and ocular axial length were significantly negatively correlated with the cross-sectional area and luminal and stromal areas of the choroid^9^. Fujiwara et al. also reported that the choroidal vascular density was related to CCT and age^10^. However, it was difficult to distinguish each tissue based on OCT pictures, and it was impossible to measure which choroidal layers were affected, such as choroidal layer thickness in normal and diseased eyes.

In a previous report examining the thickness of each choroidal layer by OCT, Branchini et al. demonstrated that the choroid of normal eyes was divided into two layers: CC plus SL, and HL^11^. Zhao et al. also manually measured each choroidal thickness in CC, SL, and HL from OCT signal features using EDI-OCT B-scan images^12^. In both studies, HL was the thickest, accounting for approximately 60 to 70% of CCT. Recently, Sonoda et al. developed a new software called "Kago-Eye2" that can semi-automatically detect the boundaries of CC, SL, and HL with high reproducibility^6^. The results of our study using Kago-Eye2, similar to previous reports, revealed that CC thickness is thinnest within the subchoroidal layer and increases from SL to HL. In addition, with Kago-Eye2, it is possible to differentiate a choroidal OCT image into a luminal area and a stromal area using a binarization method^6,13^. A feature of this study is that using our new software, we focused on the complexity and interactions of the choroidal layers and evaluated the internal structure of the choroid in detail. The results of this study revealed that the area of CC is the smallest within the subchoroidal layer, which is consistent with previous report using a conventional OCT B-scan analysis^12^. Furthermore, we added the data that the vessel diameter increased from SL to HL together with increase in the area. Histological specimens obtained from necropsy eyes showed that CC was thin, at approximately 10 μm, and SL and HL were thicker^1^. These results suggest that there is a correlation between OCT-based choroidal layer thicknesses and histological findings in this study. On the other hand, the L/C ratio was highest in CC and decreased in the order of SL and HL (*P* < 0.01). In other words, the ratio of choroidal stroma is higher in SL and HL than in CC, suggesting that melanocytes, smooth muscle, and neurons play important roles in maintaining choroidal blood flow in SL and HL.

Next, the mechanism of choroidal thinning in normal eyes is discussed. In this study, older age was associated with a smaller L/C ratio in all choroidal layers. Furthermore, a long axial length was associated with thinning of SL and HL. First, with respect to age, the large decrease in the L/C ratio of the middle and large vascular layers may be due to vessel dropout or occlusion and decreased vascular tension, changes of which are not limited to the eye but are also common in other organs with aging^14,15^. The vascular tone is affected by the circulating blood volume, BP, and endothelial nitric oxide synthase (eNOS). Because the amount of eNOS decreases with age, vascular tone subsequently decreases, and circulating blood volume reduces^14,15^. These mechanisms are likely to be involved in the decrease in the choroidal vascular lumen area on OCT images with aging. In addition, as arteriosclerosis occurs with aging and hypertension, the vascular lumen becomes vitrified and narrowed^16–18^. The present study population could potentially have included patients with well-controlled BP but pre-existing hypertension, which may have influenced the thinning of the choroid.

Second, this study revealed that choroidal thinning in the foveal region of myopic eyes is associated with a longer axial length. However, it was unrelated to CC. In enucleated eyes, the ratio of SL and HL thickness to CCT decreased with lengthening of the ocular axial^19^. On the other hand, it has been reported that CC thickness and retinal pigment epithelium (RPE) cell density in the macular region hardly change with elongation of the axial length^19^. The present study revealed a 1500-μm width at the posterior pole and so, the results might be consistent with a histology-based thickness study for each layer^19^. Mechanical posterior elongation of the eye particularly affected the vascular as well as interstitial components of HL, but not of CC. These results might contribute to preservation of the CC structures, which are essential for the functional retention of RPE.

There were some limitations of this study. First, choroidal image analysis was performed using only high-resolution horizontal single-line scans. Analysis across all quadrants of the choroid would provide more data on choroidal structures. In fact, although all measurements were done over a time range from 9:00 am to 12:00 pm, they were not strictly tailored to the time range. Third, this study had a cross-sectional retrospective design. Therefore, longitudinal studies are needed to further investigate the effects of age and axial length.

In conclusion, older age was associated with a decrease in all choroidal sublayers, and a longer axial length was associated with a decrease in the choroidal medium-large vessel layer.

## Subjects and methods

This study enrolled 189 eyes of 189 adults (82 men and 107 women, aged 59.6 ± 14.8 years old) (Table 4) who visited the ophthalmology department of Teine Keijinkai Hospital between May 2019 and December 2021.

**Table 4:** Characteristics of study subjects.

Variables	
Number of subjects, n	189
Number of eyes, n	189
Age, years	59.6 ± 14.8
Sex, male/female	82/107
BCVA-logMAR	− 0.03 ± 0.11
IOP, mmHg	14.5 ± 2.7
SE, diopters	− 2.06 ± 3.52
AL, mm	24.61 ± 1.71
SBP, mmHg	121 ± 12
DBP, mmHg	71 ± 9
CMT, μm	252 ± 19

*SD* standard deviation, *BCVA* best corrected visual acuity, *logMAR* logarithm of minimal angle of resolution, *IOP* intraocular pressure, *SE* spherical equivalent, *AL* axial length, *SBP* systolic blood pressure, *DBP* diastolic blood pressure, *CMT* central macular thickness.

We analyzed cases diagnosed as normal eyes by ophthalmologists through ophthalmoscopic examination, OCT imaging, and other ophthalmic examinations. Exclusion criteria included eyes with a history of surgery, eyes with any ocular disease other than mild cataracts, eyes with particular suspicion of age-related macular degeneration or precursor lesions such as drusen, blurred OCT images due to oculomotor abnormalities, opacification of the anterior segment/intermediate translucency, or poor fixation. This study also excluded patients with diabetic mellitus, and systemic hypertension with uncontrolled blood pressure (BP) based on medical records. In this study, the factor “age” was not set as an exclusion criterion in order to investigate the relationship between choroidal structures and age in adults.

All eyes underwent comprehensive general ophthalmic examinations including an anterior segment slit-lamp examination and fundus ophthalmoscopy. Refraction and keratometry were measured with the autorefractometer ARK-1 s (NIDEK, Aichi, Japan), and intraocular pressure with the non-contact tonometer TX-20P (Canon, Tokyo, Japan). OCT was taken with Cirrus HD-OCT 5000 (Carl Zeiss Meditec, Dublin, CA) and the ocular axial length was measured with AL-500 (Carl Zeiss Meditec, Dublin, CA) or AL-700 (Carl Zeiss Meditec, Dublin, CA). Best corrected visual acuity (BCVA) is shown as logMAR visual acuity. In addition, systolic blood pressure (SBP), diastolic blood pressure (DBP), mean BP, and the presence or absence of systemic medical diseases, were recorded. Both eyes of each subject were evaluated, but only one eye, randomly selected, was included in the statistical analysis to avoid potential bias due to interrelation between the eyes of the same subject. If a subject met exclusion criteria in only one eye, the other eye was chosen for analysis. This study was approved by the Institutional review board (IRB) of Teine Keijinkai Hospital by an opt-out method (IRB No. 2-021372-00: https://www.keijinkai.com/teine/wpadmin/wp-content/uploads/2022/02/9_OPT2-021372-00.pdf). Therefore, the need for informed consent was waived by the approving ethical committee due to the retrospective nature of the study. All testing procedures conformed to the principles of the Declaration of Helsinki.

## Choroidal stratification binarization method

**Figure 1:**
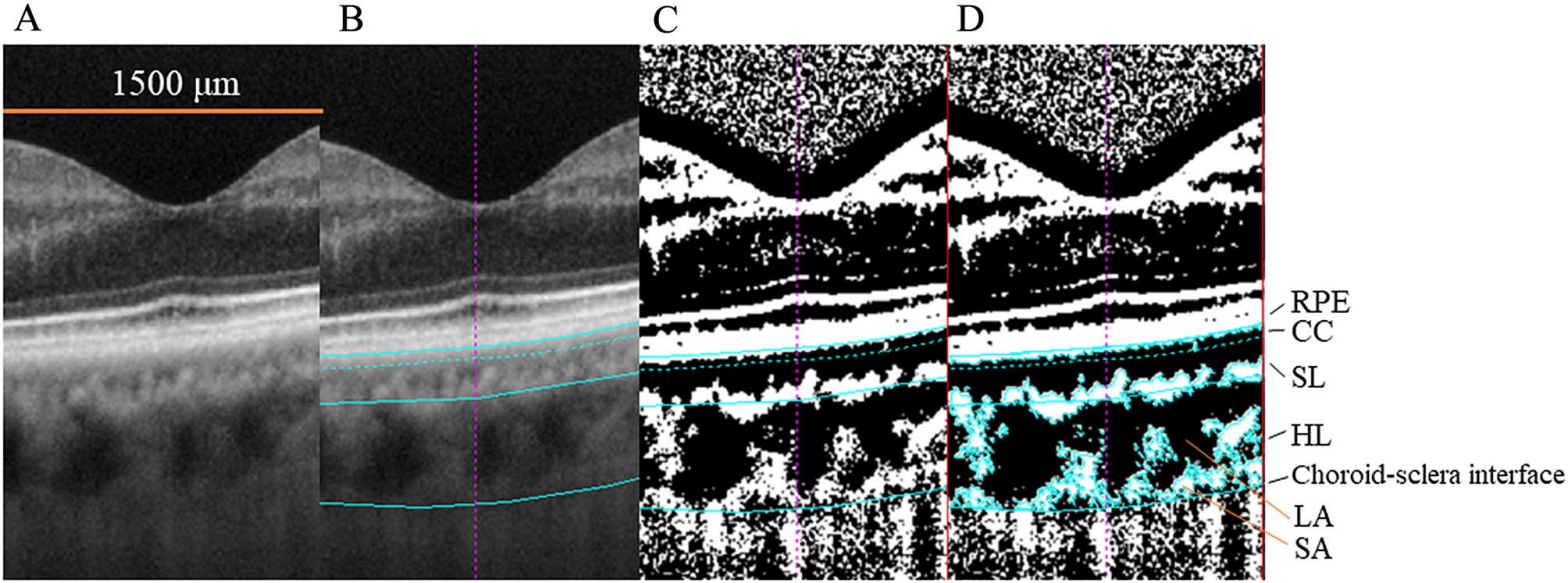
Choroidal layer binarization method. (**A**) Specify a range of 1500 μm in the EDI-OCT image passing through the fovea. (**B**) Set the lower edge of RPE, the boundary between CC and SL, the boundary between SL and HL, and the choroid and scleral boundary. (**C**) Semi-automatic binarization by Kago-Eye2 software. (**D**) Annotation of each part.

All examinations and analyzes were performed at Teine Keijinkai Hospital. Choroidal images were acquired at single horizontal HD 1 Line 100 × (100 × averaged) using an EDI-OCT eye-tracking system. The choroidal structure was analyzed using the established binarization technology^6^. Figure 1 shows a representative image of binarization of the choroid. Kago-Eye2 software is written in C +  + programming language and was created to detect the choriocapillaris-Sattler layer boundary and Sattler-Haller layer boundary in OCT images. First, the tiff image output from the OCT device was imported into the Kago-Eye2 software, and the scale of each image was adjusted by filling out the form on the screen. A square region of interest (ROI) was determined on the screen, and the left and right borders of ROI were aligned to identify a 1500-μm-wide choroidal region centered on the fovea. The evaluator then manually selected three representative vessel lumens that were the darkest in Haller’s layer of the OCT image. The average reflectance of these three lumens is expressed as a brightness tone and was used as a criterion for the lowest brightness cut value. Using these data, the cut out image was converted into a new image with 256 gradations (0 to 255). The image was binarized using the binarization threshold obtained in the above step. Define the boundary between the choriocapillaris and Sattler’s layers: Plot the brightness data from the RPE layer to the choroidal–scleral interface. These data are placed in a rectangle of uniform height, and the average brightness of pixels on the same horizontal line is calculated. Second derivatives are then used to analyze changes in these means. Specifically, changes in the average value are analyzed using second-order differentiation, and pixels that meet specific conditions are set as the choroidal boundary. As a rule, the OCT reflection intensity is dark in the luminal area and bright in other area. Using this principle, we assume that the luminal component changes from choriocapillaris to the Sattler’s layer across the stromal area, plot changes in reflection intensity vertically, perform differentiation, and search for major trend change points. The change points are then arranged in pixels in the horizontal direction, and the change points are plotted and connected to draw an approximate line. Defining the boundary between Sattler's and Haller's layers: Extract brightness data for each pixel in the vertical direction from the RPE layer to the boundary between choriocapillaris and Sattler's layer. It then detects the vertical brightness changes in the image and identifies the boundary points. This identifies the boundaries between choriocapillaris and Sattler's layer and between Sattler's and Haller's layers. Additionally, these boundaries are fitted with quadratic curves to define the boundaries. When defining choroidal boundaries, we perform segmentation using a combination of clear numerical data and image analysis, resulting in more objective and reproducible results. Finally, the obtained binarization threshold was used to binarize the OCT images of the choroidal area, which included the choroidal area (CA), luminal area (LA), and stromal area (SA), and the central choroidal thickness (CCT) was automatically measured. Bright and dark pixels corresponded to SA and LA, respectively. The ratio of LA to CA was defined as the L/C ratio.

## Statistics

**Figure 2:**
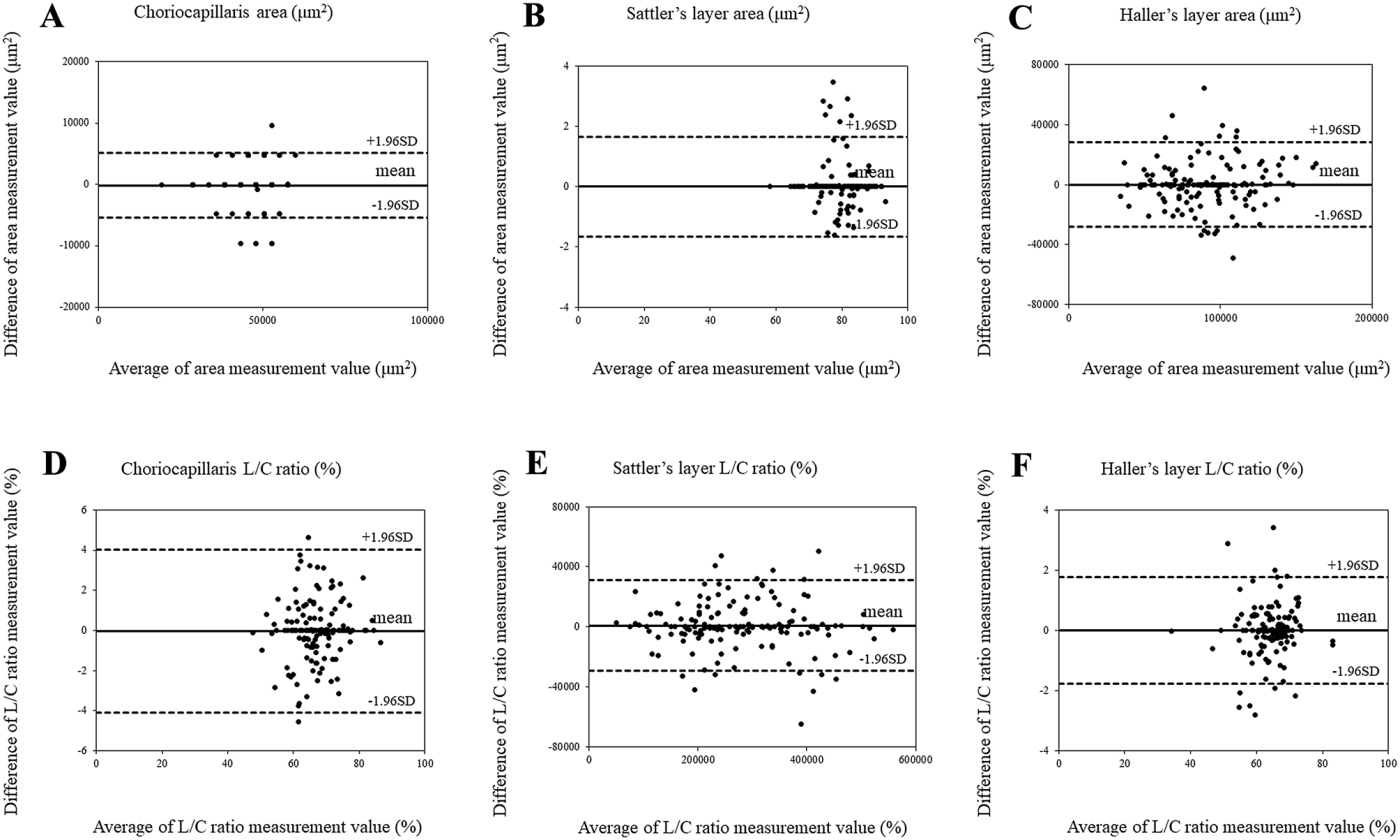
Agreement between the choroidal area and luminal/choroidal area ratio (L/C ratio) using Bland–Altman plot. (**A**) Choriocapillaris area in normal eyes. (**B**) Sattler’s layer area in normal eyes. (**C**) Haller’s layer area in normal eyes. (**D**) Choriocapillaris L/C ratio in normal eyes. (**E**) Sattler’s layer L/C ratio in normal eyes. (**F**) Haller’s layer L/C ratio in normal eyes.

All images of binarization were obtained by two examiners. In the case of any discrepancies, the senior author decided which measurement was correct. In addition, Bland–Altman analysis was performed to check reproducibility and reliability, and the mean value was used (Fig. 2). Statistical analysis was performed using commercially available statistical software SPSS version 21 (IBM Corporation, Chicago, USA). Spearman's rank correlation coefficient was used for statistics (Table 1). Multiple regression analysis was performed for choroidal parameters, age, refractive power, and ocular axial length in each choroidal layer. The objective variables were choroidal structure parameters, and the explanatory variables were age, refraction, and ocular axial length. The Kruskal–Wallis test was used to compare choroidal areas by choroidal layers and examine the L/C ratio in each choroidal layer (Supplementary Information).

### Supplementary Information

Supplementary Information 1.

Supplementary Information 2.

## Data Availability

The datasets used and/or analyzed during the current study are available from the corresponding author on reasonable request.
